# 592. Antimicrobial Utilization in Solid Organ Transplant Recipients 12-Months Post-Transplantation

**DOI:** 10.1093/ofid/ofab466.790

**Published:** 2021-12-04

**Authors:** Tommy J Parraga Acosta, Sage Greenlee, Charles Makowski, Rachel Kenney, Ramesh Mayur, George J Alangaden

**Affiliations:** Henry Ford Hospital, Dearborn, Michigan

## Abstract

**Background:**

Antimicrobials are widely used in solid organ transplant recipients (SOTr). Yet, antimicrobial utilization in the transplant (TP) population is not well characterized. National Healthcare Safety Network antimicrobial use (NHSN-AU) does not provide data specific to SOTr. This study sought to describe inpatient antibiotic use among SOTr up to 1-year post-TP.

**Methods:**

A cross-sectional study was performed of all SOTr who received a TP between January 2015 to December 2016. Demographics, TP type, antibiotic use variables, hospital days, and *Clostridioides difficile* infection (CDI) are described. Inpatient antibiotic administration was measured for 365 days starting from date of TP surgery. Automated data generated for NHSN-AU reporting was utilized, and SOTr data was abstracted by cross-matching with the transplant database. Transplant-patient days was used as the denominator for metrics. Variables included duration of therapy (DOT), DOT/1000 patient days, antimicrobial free days (inpatient days no antimicrobials were administered), and NHSN-AU reporting targets of anti-methicillin resistant S. aureus (MRSA), broad spectrum, and high-risk CDI agents. Data was analyzed using descriptive statistics via Microsoft Excel®.

**Results:**

A total of 530 SOTr were analyzed. Baseline characteristics are shown in Table 1. Median age was 61, male gender 64%, median Charlson Comorbidity Index was 5. Kidney TP (43%), liver TP (32%), lung (9%) and heart (8%) were most common TP types. Among these four TP types: Lung TP had the highest median DOT (13 days), DOT/1000 patient days (6.6) and ratio of DOT/total patient (1.9) (Table 2). Liver TP had the most antimicrobial free days (34%). Proportionally, anti-MRSA agents use was highest in thoracic TP (lung/heart), broad-spectrum agent use was common in all but kidney TPs, and high-risk CDI agents use was highest among kidney TP (Table 3). A total of 34 SOTr had CDI, 76% in kidney/liver TPs.

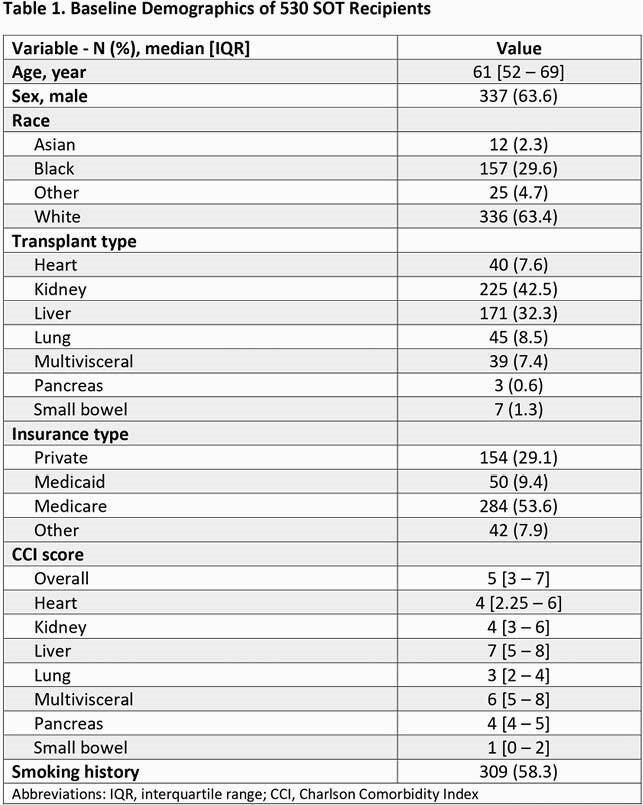

Table 1. Antimicrobial usage and SOT - ID Week 2021

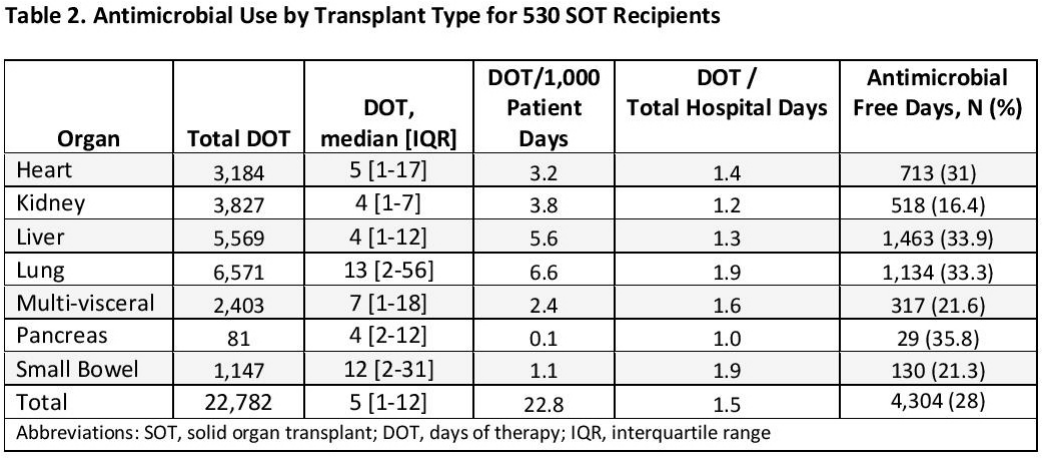

Table 2. Antimicrobial usage and SOT - ID Week 2021

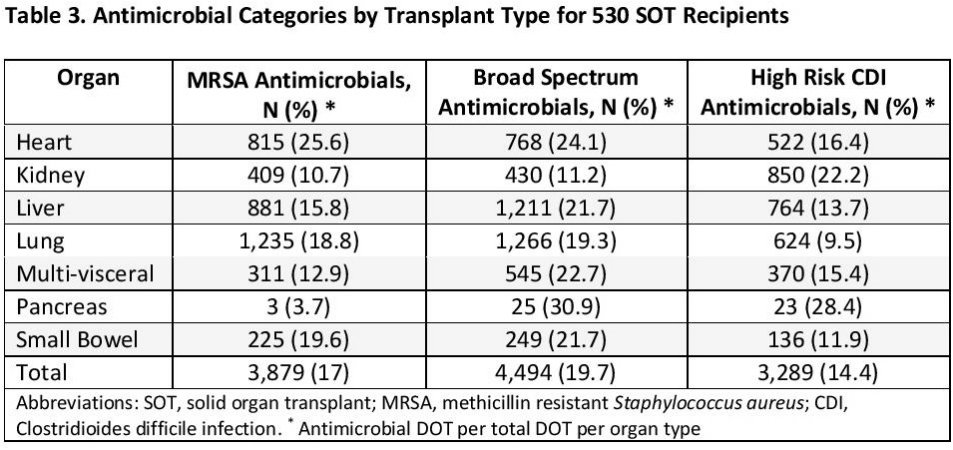

Table 3. Antimicrobial usage and SOT - ID Week 2021

**Conclusion:**

Our study provides preliminary and important data of inpatient antibiotic utilization specifically in SOTr, generated using automated NHSN-AU data cross-matched to transplant database. These metrics can be utilized to promote antimicrobial stewardship efforts directed to specific TP types.

**Disclosures:**

**Rachel Kenney, PharmD**, **Medtronic, Inc.** (Other Financial or Material Support, spouse is an employee and shareholder)

